# Frequency of safety signals from scientific reports, manufactures, registers, and other sources for a random selection of hip and knee prostheses

**DOI:** 10.2340/17453674.2025.44035

**Published:** 2025-06-25

**Authors:** Yijun REN, Lotje A HOOGERVORST, Enrico G CAIANI, Perla J MARANG-VAN DE MHEEN, James A SMITH, Alan G FRASER, Rob G H H NELISSEN, Anne LÜBBEKE

**Affiliations:** 1 Department of Electronics, Information and Biomedical Engineering, Politecnico di Milano, Milan, Italy; 2 Department of Orthopaedics, Leiden University Medical Center, Leiden, the Netherlands; 3 Istituto Auxologico Italiano IRCCS, Milan, Italy; 4 Safety & Security Science and Centre for Safety in Healthcare, Delft University of Technology, Delft, the Netherlands; 5 Botnar Research Centre and Centre for Statistics in Medicine, Nuffield Department of Orthopaedics, Rheumatology and Musculoskeletal Sciences, University of Oxford, Oxford, UK; 6 National Institute for Health Research Oxford Biomedical Research Centre, John Radcliffe Hospital, Oxford, UK; 7 Department of Cardiology, University Hospital of Wales, Heath Park, Cardiff, UK; 8 Division of Orthopaedic Surgery and Traumatology, Geneva University Hospitals and University of Geneva, Geneva, Switzerland; 9 Nuffield Department of Orthopaedics, Rheumatology and Musculoskeletal Sciences, University of Oxford, Oxford, UK

## Abstract

**Background and purpose:**

The safety and performance of hip and knee prostheses can be assessed by analyzing peer-reviewed literature, registry reports, and safety notices published by national competent authorities/regulatory agencies, or manufacturers. The percentage of hip and knee prostheses with a safety signal published through any of these data sources is unknown. We aimed to assess the frequency of signals identified for a random sample of 10 hip stems, 10 hip cups, and 10 knee implants.

**Methods:**

3 literature libraries were searched to find safety signals defined as information on patterns/occurrences that may alter the device’s benefit–risk profile, reported in peer-reviewed publications for the randomly selected implants. Annual registry reports from 5 national registries were examined to check whether any of the selected implants had outlier performance. The CORE-MD post-market surveillance (PMS) tool was used to collect all related safety notices from 13 competent authority/regulatory agency websites. Manufacturers’ websites were screened for any reported safety information.

**Results:**

Safety signals were identified for 21 of the 30 randomly selected implants: 18 identified by registries, 7 by the CORE-MD PMS tool, and 8 based on literature, with 10 implants identified by multiple sources. There was no systematic pattern in timing of publication with a particular source publishing safety signals earlier than other sources.

**Conclusion:**

70% of the randomly selected hip and knee prostheses had ≥ 1 safety signals published, with registries as the source for the majority. No single source identified all 21 implants with signals, which highlights the need for a comprehensive surveillance strategy to aggregate safety signals from multiple sources.

Hip and knee prostheses are deemed to be the most successful treatment for severe degenerative osteoarthritis [1], but failures related to prostheses still occur [2,3]. A prominent example relates to metal-on-metal total hip arthroplasty (THA) [4,5]. Registry data showed much higher revision and mortality rates than for other comparable THA [4,6]. Consequently, safety concerns regarding this finding were internationally circulated, but only after > 1 million patients had received them [7]. To minimize harm, it is important to monitor the performance of implants and promptly share safety signals internationally with clinicians, patients, and regulators [8].

Signal detection is defined by the International Medical Device Regulators Forum (IMDRF) as the process of identifying patterns/occurrences that may alter the device’s benefit–risk profile [9]. There are 4 main data sources to identify safety signals, each with unique strengths and weaknesses: scientific literature, registry reports, manufacturers’ websites, and national competent authorities’/regulatory agencies’ websites. Scientific literature reports clinical and patient-reported outcomes and surrogate endpoints (e.g., imaging), potentially providing early safety signals [10], but studies may have limited sample sizes and therefore be less useful for evaluating revision risks. Registries evaluate all-cause revision to identify implants with outlier performance (i.e., having a significantly higher revision risk than other comparable implants), while recognizing that revision risk is influenced by factors such as patient, disease, and surgical characteristics besides the implant performance [10]. Moreover, the criteria for identifying outliers differ between registries, for example having a prosthesis time-incident rate (PTIR) more than twice the PTIR of the group [11] or a revision rate > 1.3 per 100 observed component years [12]. Thus an implant may be identified in 1 registry, but not in another [13]. Additionally, in countries with generally low revision rates, smaller absolute differences between implants in revision risks could also result in an implant being identified as an outlier in 1 country but not in others. Safety notices, available on the websites of manufacturers or competent authorities/regulatory agencies, assess a wider variety of implant-related issues (e.g., packaging and labelling). Knowledge regarding the most informative data source for highlighting implants with safety signals is still limited.

We aimed to assess the frequency and content of safety signals identified across these 4 data sources for a random sample of 10 hip stems, 10 hip cups, and 10 knee implants.

## Methods

### Study design

**Figure 1:**
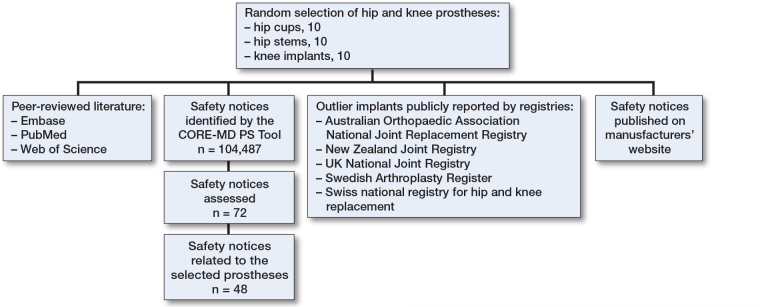
Schematic overview of the study design and data sources.

This observational study was designed to describe safety signals reported for a random selection of hip and knee prostheses in existing real-world data sources. The study is reported according to STROBE guidelines (Figure 1).

### Random selection of hip and knee prostheses

The process of selecting the random sample set of hip and knee prostheses has been published previously [14]. Briefly, to create a sample of Conformité Européenne (CE)-marked implants, all implants reported on the website of the Orthopaedic Data Evaluation Panel (ODEP) and 8 European national arthroplasty registries (Denmark, Finland, Germany, the Netherlands, Norway, Sweden, Switzerland, and the National Joint Registry [NJR]) were used, resulting in a list of 138 hip cups, 165 hip stems, and 97 knee implants. rom this list, 10 hip cups, hip stems, and 10 knee implants were randomly extracted (Table).

**Table:** The randomly selected implants with the corresponding manufacturer

Hip stem
Accolade II (uncemented, Stryker)
Alloclassic Zweymuller SL (uncemented, Zimmer)
Avenir (uncemented, Zimmer)
BiContact (uncemented, B Braun)
C-stem AMT Total Hip System (cemented, DePuy)
COLLO-MIS (uncemented, LimaCorporate)
Filler 3ND (cemented, Biotechni)
MiniHip (uncemented, Corin)
QUADRA (uncemented, Medacta)
Stelia (uncemented, Stemcup)
Hip cup
ANA.NOVA (uncemented, ImplanTec)
AneXys (uncemented, Mathys)
Cenator (cemented, Corin)
EcoFit (uncemented, Implantcast)
Exceed ABT (uncemented, Zimmer)
IP X-LINKed (cemented, Waldemar LINK)
Plasmacup SC (uncemented, B Braun)
POLARCUP (cemented, Smith & Nephew)
RM pressfit Vitamys (uncemented, Mathys)
Versafit CC Trio (uncemented, Medacta)
Knee implant
ACS Unicondylar (uncemented, Implantcast)
BalanSys CR (Mathys)
Innex Gender (Zimmer)
LCS complete (DePuy)
NexGen CR (Zimmer Biomet)
Optetrak CR (Exactech)
Optetrak Logic RBK (Exactech)
Sigma High Performance Partial Knee (DePuy)
TREKKING CR (SAMO)
Vanguard CR (ZimmerBiomet)

### Identifying safety signals in peer-reviewed literature

3 literature libraries (Embase, PubMed, and Web of Science) were searched for publications in English, German, and French to include all studies reporting clinical investigations on the implant of interest [14]. No journal restrictions were applied. Safety signals were included across a range of outcomes: (i) all-cause revision; (ii) implant migration; (iii) periprosthetic osteolysis; (iv) patient-reported outcome measurements, and (v) postoperative orthopedic complications relevant to the prostheses (14).

### Identifying safety notices published on websites of national competent authorities/regulatory agencies

Safety notices were defined by the Medical Device Regulation as communications sent by manufacturers to users/customers regarding corrective actions [15]. The process to identify and categorize safety notices has already been described [16]. Briefly, for each selected implant, the Coordinating Research and Evidence for Medical Devices (CORE-MD) post-market surveillance (PMS) tool [17], an automated web scraper tool, was used to extract all safety notices from the national competent authorities’/regulatory agencies’ websites in 13 countries. We refer to safety notices to indicate the collective safety information found on these websites, including alerts and recalls.

Each extracted safety notice was then manually evaluated and the reported events were categorized based on the extended description using the IMDRF codes [18]. Given the hierarchical structure of the IMDRF codes, categorization was conducted to the most detailed level available, independently by YR and LH; possible differences in coding were resolved through discussion.

Duplicate safety notices could occur if national competent authorities/regulatory agencies from different countries issued identical safety notices that they had received from the same manufacturer about the same implant concerning the same problem. Each safety notice was manually analyzed and identified duplicates were excluded from further analysis.

### Identifying safety information published on manufacturers’ websites

As manufacturers are responsible for providing information on the safety and performance of their implants, the websites of the manufacturers were screened, to assess whether they had published any safety notice(s) related to each selected implant.

### Identifying implants with significantly higher revision risks in registries

To identify registries publicly reporting on implants with significantly worse performance than other implants (i.e., outlier implants; having significantly higher revision risks), we used the same method as previously described [16]. Only national registries that publicly reported implant outliers were used for the safety signal assessment [13,19]: Australian Orthopaedic Association National Joint Replacement Registry (AOANJRR), New Zealand Joint Registry (NZJR) [20], UK NJR, Swedish Arthroplasty Register (SAR) [21], and Swiss national registry for hip and knee replacement (SIRIS). All their available annual reports were screened for content relating to each selected implant, based on brand name. We documented whether the selected implant was identified as an outlier itself or as part of an outlier cup–stem combination.

### Timing of publication of safety signals

Safety signals were included from all 4 data sources until December 31, 2022. To assess whether some data sources systematically reported earlier, we extracted the publication dates of safety signals for each implant and data source.

### Funding, use of AI tools, and disclosures

This work was supported by the European Union’s Horizon 2020 Research and Innovation Programme (grant number 965246) and was part of the CORE-MD project.

AI tools were not used.

Complete disclosure of interest forms according to ICMJE are available on the article page, doi: 10.2340/17453674.2025.44035

## Results

### Safety notices from peer-reviewed literature and registries

**Figure 2:**
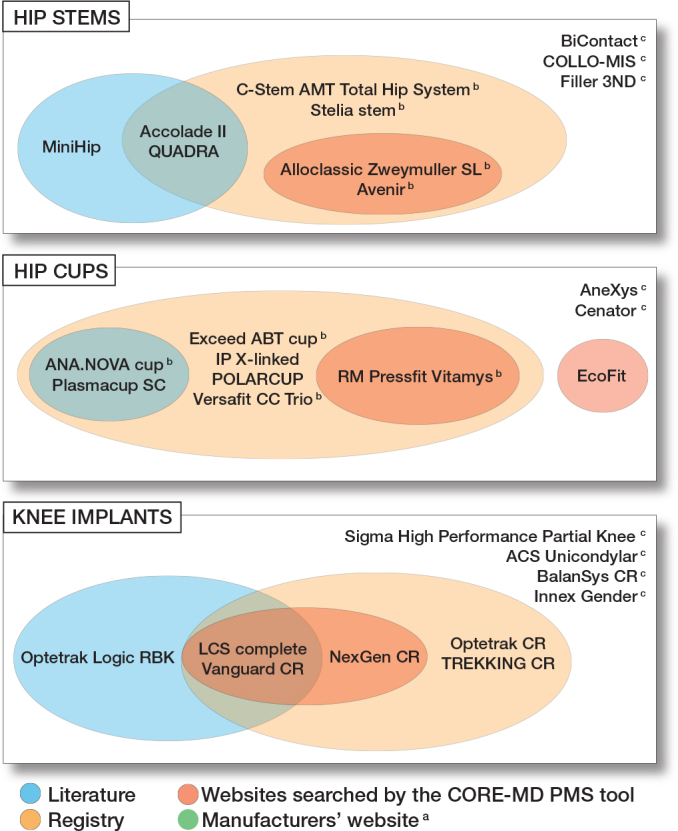
Frequency of safety signals across the 4 data sources. ^a^ No safety signal was found on the websites of the manufactuers for any of the selected implants. ^b^ Identified only in combination with a specific other implant (see Supplementary Tables 1–2). ^c^ No safety signal was found on any sources.

For the 30 randomly selected implants, 16 safety signals were identified from the literature relating to 8 of the implants, and 23 safety signals were identified through the registries’ outlier identification procedures relating to 18 of the implants. For the hip implants, 10 out of 12 safety signals were released for a cup–stem combination that included the selected implant. Regarding knee implants, 3 were potential outliers (labelled “maybe”), and 2 were confirmed outliers (Supplementary Tables 1–3). In 6 implants (2 stems, 2 cups, and 2 knee implants) an overlap of safety signals was found between literature and registry reports (Figure 2).

### Safety notices from the websites of national competent authorities/regulatory agencies

Of 104,487 safety notices published up to December 31, 2022 on websites of national competent authorities/regulatory agencies, 72 safety notices relating to the 30 implants were extracted, based on manufacturer and implant brand name. 24 safety signals were excluded from further analysis as they did not relate to the specific implant (but instead to surgical kits or a different variant of the brand name), thus resulting in 48 safety notices to be included for further analysis (Supplementary Figure 1).

The majority of safety notices (31 out of 48) originated from the USA (Supplementary Figure 2), where separate safety notices were issued for various parts, sizes, and variants of the same brand of an implant. Removing duplicates resulted in 17 safety notices corresponding to 7 unique implants: 2 hip stems, 2 hip cups, and 3 knee implants (see Supplementary Tables 1–3). The majority of them (8 out of 17) reported medical device problems related to the IMDRF-code “A210106 – wrong label” (Supplementary Table 4).

### Random set of hip stems

Of the 10 randomly selected hip stems, 3 did not have any safety signals published across any of the 4 data sources (see Figure 2 and Supplementary Table 1). 1 hip stem had safety signals published solely in the peer-reviewed literature (implant breakage, poor patient-reported clinical outcomes [22]); 2 were identified solely by registries as an outlier implant; 2 were identified both by registries and by the CORE-MD PMS tool (IMDRF code “wrong label”); and 2 were identified by both the peer-reviewed literature [23-25] and by registries, although in one case the safety signal described in the literature was not related to the implant itself, but to a surgery-related issue. No hip stem was identified by all 4 data sources, and none had a safety signal published on its manufacturer’s website.

### Random set of hip cups

2 hip cups did not have any safety signals published across the 4 data sources. 4 hip cups were identified as an outlier solely by registries; 2 by both registries and the peer-reviewed literature (increased total migration [26], poor patient-reported clinical outcomes [27,28], osteolysis [29], and ceramic head fracture [28,30]); 1 by both registries and the CORE-MD PMS tool (“difficult to open or remove packaging material”); and 1 solely by the CORE-MD PMS tool (“detachment of device or device component” and “inadequate instructions for healthcare professional”) (see Figure 2 and Supplementary Table 2). No hip cup was identified in all 4 data sources and none had safety signals published on its manufacturer’s website.

### Random set of knee implants

Of the 10 randomly selected knee implants, 4 implants did not have any safety signals published across the 4 data sources (see Figure 2 and Supplementary Table 3). 2 knee implants had safety signals published in the literature (fracture through the polyethylene insert [31], poor patient-reported clinical outcome [32], higher risk of aseptic tibial loosening [33], and higher risk of early revision [34]) and were identified using the CORE-MD PMS tool (“improper or incorrect procedure or method,” “defective component,” and “wrong label”) as well as identified by registries as outlier implants. 2 implants were identified only by registries, and 1 solely by literature [2,3]. None of the 10 knee implants had safety notices published by all 4 data sources and none had safety signals published on its manufacturer’s website.

### Analysis by data source and timing

Across all randomly selected implants with safety signals, registries were the most frequent data source of safety signal detection, identifying 18 of the 21 implants with any safety signals. Peer-reviewed literature and the CORE-MD PMS tool added safety signals for 2 implants and 1 implant, respectively, that were not identified by other data sources. In total, 10 implants had safety signals generated by multiple data sources (Supplementary Table 5). Registries provided the first signal for 3 implants (1 knee implant and 2 cup–stem combinations), published literature for 3 implants, and safety notices for 3 implants, indicating that no data source systematically published safety signals earlier than other sources.

## Discussion

70% of the randomly selected hip and knee implants had been the subject of at least 1 safety signal. Of the 4 data sources, and for clinical insights, registries were the most informative as they identified most implants with safety signals. No safety information was found on its manufacturer’s website for any of the implants. The other data sources (i.e., peer-reviewed literature and the CORE-MD PMS tool) each identified implants that had not been reported as outliers by registries.

Although the chance of identifying an implant with a safety signal is highest when assessing registry data, signal detection is then based solely on revision risks. Hence, different data sources are needed to capture safety signals that occur earlier than revision and concern other clinical variables, such as patient-reported outcome measures or surrogate indicators obtained by imaging.

Different safety signals were identified, from labeling errors (i.e., Avenir) to contamination problems (i.e., NexGen CR), all of which could potentially lead to recalls (i.e., the removal or correction of a marketed product [35]). Additionally, we analyzed manually the FDA medical device recall website [36], the only regulatory agency systematically publishing such information, for the 16 implants that were FDA-approved [14]. 7 FDA recalls were found, of which 5 were already identified in our analysis and 2 were newly identified safety signals (Optetrak CR and Optetrak RBK). This demonstrates that reviewing diverse sources effectively captures most recall-related issues but also highlights the complexity of comprehensive PMS.

### Comparison with previous studies

In a recent study, we identified 47 unique total knee prostheses that had either outlier performance identified by registries or safety notices published by national competent authorities/regulatory agencies [16]. Of these, 55% had been the subject of both safety notices and outlier performance, 26% had been named only in safety notices, and 19% had been identified only as outliers. Our earlier study suggested that safety notices and registry outliers measure different aspects of implants’ safety and performance [16]. However, in that study, we did not analyze which part of total knee prostheses did not have any safety signals, as it took the published safety signal as entrance for the study rather than considering all implants at risk. It would be useful to consider and compare all data sources of post-market clinical data for all implants at risk, but, given the enormous number of implants available on the market across all countries, such analysis would be feasible only for a random selection of implants. This study was therefore designed to complement our previous findings by adding manufacturers’ websites and the peer-reviewed literature as data sources, and extending the enquiry by also including hip prostheses. If a data source consistently publishes safety signals earlier than others, then it should be known as the best data source for early warning. Otherwise, a comprehensive surveillance strategy that integrates all data sources to detect safety signals as early as possible would be needed.

### Content and significance of safety signals

Different types of medical device problem codes were identified. Some were more likely to influence risk of revision, such as “defective component” for the Vanguard CR knee implant. Others would have less or even no influence on revision, such as “difficult to open or remove packaging material” for the RM Pressfit Vitamys hip cup. The first of these implants, the Vanguard CR, had a defective component mentioned in its safety notice, and also a higher revision risk identified both in registries and in the literature, so all data sources likely pointed to the same underlying problem. For the second case, the RM Pressfit Vitamys hip cup was identified by registries as having an outlier performance, but exclusively when it was combined with a cemented CCA hip stem; so the problem is more likely to be associated with the particular cup–stem combination, or even with the stem alone. Intuitively, the problem reported as “difficult to open or remove packaging material” is less likely to influence risk of revision, but it could affect the duration of surgery. These examples show that when different data sources identify safety signals related to the same implant, they may indicate either the same underlying condition (e.g., the Vanguard CR knee implant) or separate issues for the same implant/combination (as shown in the case related to the RM Pressfit Vitamys hip cup).

Safety signals reported in the literature included factors that are likely to affect revision rates, such as implant breakage, aseptic loosening, increased implant migration, and osteolysis. However, of the 7 implants to which these safety signals referred (1 implant had only a surgery-related issue), only 4 were also identified by registries as having outlier performance. This may be due to: (i) patient factors affecting the risk of revision, such as a high BMI [37,38]; (ii) indicators used to compare outcomes in the literature differing from the indicators used in registries; (iii) the study reporting on an institution that was an early adopter of the implant and the registry has not yet identified the implant as an outlier; (iv) the (hip) implant combination not being the same in the study as in the registries, or (v) a reported safety signal being based on an outcome other than revision (e.g., Quadra stem [24,25]).

While registries remain invaluable for highlighting early signals of potentially failing implants, they cannot establish causality, making clinical studies essential to identify the underlying causal mechanism. A relevant example is the Continuum cup (Zimmer Biomet), where concerns regarding increased risk of early revisions were flagged by the AOANJRR and confirmed by a registry-based study from Finland [39]. However, a clinical study was needed to identify the root cause of insufficient coverage of the neutral liner, resulting in higher dislocation rates [40].

### Responsibilities of manufacturers

To the best of our knowledge, no previous study has examined medical device manufacturers’ websites for the extent to which their safety information was fully (publicly) reported. A comparable examination has, however, been done for the websites of the 44 biggest pharmaceutical companies [41]; only about half of the pharmaceutical websites reported all effects related to drugs with a high incidence of side effects (> 10%), and therefore it was concluded that pharmaceutical companies were unlikely to communicate complete information regarding risks. In our study, there were 16 different manufacturers of the selected implants, and none published any safety information concerning their implants on their websites.

Manufacturers of high-risk implanted devices should not only prepare and make easily accessible the summaries of safety and clinical performance for their implants, which are now legally required in the European Union, but they should also provide for patients and doctors details of implant alerts and safety notices and any information from registries on outlier performance. When posting all this information on their websites, they can categorize reports that are minor, while highlighting any issues that indicate a need for increased clinical vigilance.

### Limitations

We chose to select implants randomly, as that could increase the generalizability of our findings to other hip and knee prostheses on the market, but some limitations should be noted. First, not all data sources described implants unambiguously (i.e., using a unique device identifier), leading possibly to an overestimation of the proportion of implants with safety signals. We have highlighted this in the Supplementary data, by indicating if reports concerned specific implant combinations, and by labelling as “maybe” any safety signal when the implant description was too imprecise. Second, the CORE-MD PMS tool searched for safety notices published by 13 national competent authorities/regulatory agencies. We may have missed some relevant notices published only by other authorities. A third limitation arises from the keywords (i.e., manufacturer and brand names) used for querying the CORE-MD PMS tool. While this could ensure high accuracy in identifying true positives (i.e., relevant safety notices correctly identified), it may also generate false negatives (i.e., missing relevant safety notices) due to incomplete information in safety notices. Fourth, the search strategy was designed to identify all safety signals published in the peer-reviewed literature, related to the implants of interest [14], but it could still have missed some relevant publications, for instance in languages other than English, German, or French. Fifth, our analysis focused on safety signals regarding tibial/femoral components of knee implants, excluding liner-related factors (e.g., liner malseating and modularity), which are well-documented causes of liner-associated failures [42,43]. Consequently, this may have resulted in an underestimation of safety signals for these implants.

### Conclusion

70% of a randomly selected group of hip and knee prostheses on the market had 1 or more safety signals published across several data sources. Registries reported the majority of implants with safety signals, meaning that the problems translated to a higher risk of revision of the implant (alone or in a given combination) than comparable implants. Given that safety signals may also relate to outcomes other than revision, a multifaceted approach analyzing all 4 data sources is needed. Precise implant identification through a unique device identifier used by all data sources is crucial for better and faster detection of safety signals.

### Supplementary data

Supplementary Tables 1–5 and Supplementary Figures 1–2 are available as Supplementary data on the article homepage: doi: 10.2340/17453674.2025.44035

## Notes

YR, LH: data collection, statistical analysis, interpretation of data, writing the original draft of the manuscript. EGC, PJM-vdM, JAS, AGF, RGHHN: critical revision of the manuscript for important intellectual content. AL: data collection, interpretation of data, critical revision of the manuscript for important intellectual content.

Handling co-editors: Keijo Mäkelä and Robin Christensen

*Acta* thanks Antti Eskelinen and other anonymous reviewers for help with peer review of this manuscript.
